# Translational Evaluation of an Intraparenchymal Collagen Matrix Tamponade: Initial Preclinical and Clinical Experiments to Prevent CSF Reflux Following Endoscopic Brain Surgery

**DOI:** 10.3390/ijms26189081

**Published:** 2025-09-18

**Authors:** Yasuo Aihara, Kentaro Chiba, Yuichi Oda, Kevin Browne, Dmitriy Petrov, Takakazu Kawamata, John C. O’Donnell

**Affiliations:** 1 Department of Neurosurgery, Tokyo Women’s Medical University, Tokyo 162-8666, Japan; chiba.kentaro@twmu.ac.jp (K.C.); oda.yuichi@twmu.ac.jp (Y.O.);; 2 Center for Brain Injury & Repair, Department of Neurosurgery, Perelman School of Medicine, University of Pennsylvania, Philadelphia, PA 19104, USA; kbrowne@pennmedicine.upenn.edu (K.B.); Dmitriy.Petrov@pennmedicine.upenn.edu (D.P.); odj@pennmedicine.upenn.edu (J.C.O.); 3 Center for Neurotrauma, Neurodegeneration & Restoration, Corporal Michael J. Crescenz Veterans Affairs Medical Center, Philadelphia, PA 19104, USA

**Keywords:** cerebrospinal fluid leak, DuraGen, regenerative healing, inflammation, neuroendoscope, parenchymal tract

## Abstract

Transparent polymer sheaths are often utilized in neuroendoscopic procedures to minimize intraventricular bleeding and parenchymal injuries. However, cerebrospinal fluid (CSF) leakage remains a common complication following neuroendoscopic surgery for intraventricular and deep-seated lesions. We investigated an innovative technique to prevent postoperative CSF leakage through the tract using a collagen matrix dural graft. A rolled collagen matrix (DuraGen^®^) was used as a parenchymal tract tamponade to seal the tract created by an angiocatheter (preclinical pilot) or neuroendoscopic sheath (clinical case studies). A small pilot study using a juvenile pig model was first conducted to test the implantation technique and to evaluate the inflammatory response to, and absorption of intraparenchymal DuraGen. The efficacy of this approach was then assessed in two clinical cases using MRI at postoperative days 1, 7, 40, and 60. The outer segment of the graft was unfurled to cover the dural defect for clinical application. In the pig model, histological analysis showed healing with minimal inflammation in DuraGen^®^-implanted hemispheres, while untreated control tracts exhibited parenchymal scarring and chronic inflammation. In both patients, postoperative MRI demonstrated resolution of subdural fluid collections and progressive absorption of DuraGen^®^ with no complications. This technique ameliorated CSF leakage and enhanced parenchymal healing after neuroendoscopic surgery. DuraGen^®^ may modulate the local environment for tissue repair beyond its use in dural grafting.

## 1. Introduction

The development of minimally invasive surgical approaches to deep-seated structures has dramatically changed neurosurgical practice and improved outcomes. However, the repeated introduction of an endoscope can cause intraventricular bleeding and parenchymal injuries. Transparent peel-away or other sheaths have been developed as working channels for the neuroendoscope and surgical instruments [1,2]. When inserted in the ventricle, the sheath protects brain parenchyma and provides a clear pathway for neuroendoscopic procedures [3]. However, challenges persist, particularly in cases where postoperative complications such as subdural cerebrospinal fluid (CSF) collection and CSF leakage through dural defects are common after neuroendoscopic procedures [4,5,6]. Removing the sheath results in pooling of CSF from the ventricle, filling the trajectory with fluid and overflowing through the dural opening [7]. Hemostatic agents may be used to pack the tract to prevent CSF leaks [7,8,9]; however, this technique does not always work as desired and is often followed by inflammation along the walls of the parenchymal tract. Various dural grafts have been reported for duraplasty within the burr hole, but there is a paucity of reports on packing the parenchymal tract itself to prevent CSF effusion and leakage.

The collagen matrix dural substitute graft DuraGen (Integra LifeSciences, Princeton, NJ, USA) has been widely used for dural repair in neurosurgery since 1999 in the US and 2019 in Japan. DuraGen is placed either beneath the dura mater (“inlay”) and/or over it (“onlay”) to prevent CSF leak and promote dural repair [8]. This report introduces a novel technique using DuraGen to prevent CSF leakage in neuroendoscopic procedures and explores its plausible role in promoting parenchymal healing. We describe a patient undergoing neuroendoscopic surgery in whom a DuraGen sheet was rolled into a cylindrical shape to fill the brain parenchymal defect created by the sheath insertion. This method effectively protected against subdural CSF accumulation, CSF leakage from the burr hole, and subcutaneous fluid collection. We also observed minimal inflammation and enhanced healing in the tract filled with DuraGen based on MRI findings. This approach was inspired by an animal pilot study in which DuraGen inserted into a cortical parenchymal tract in a porcine model showed marked attenuation of the inflammatory response and a pro-regenerative effect on parenchymal healing, compared to controls where the cortical tract remained empty.

The concept of using a cylindrical collagen matrix plug to prevent CSF leakage originated from clinical concerns about tract-related fluid collection after neuroendoscopic procedures. This prompted the development of a dedicated preclinical study using a porcine model, which confirmed the feasibility, sealing effectiveness, and biocompatibility of the DuraGen-based technique. Encouraged by these findings, we proceeded to apply this approach in a selected neuroendoscopic surgery case.

## 2. Results

### 2.1. Case Report

**Figure 1:**
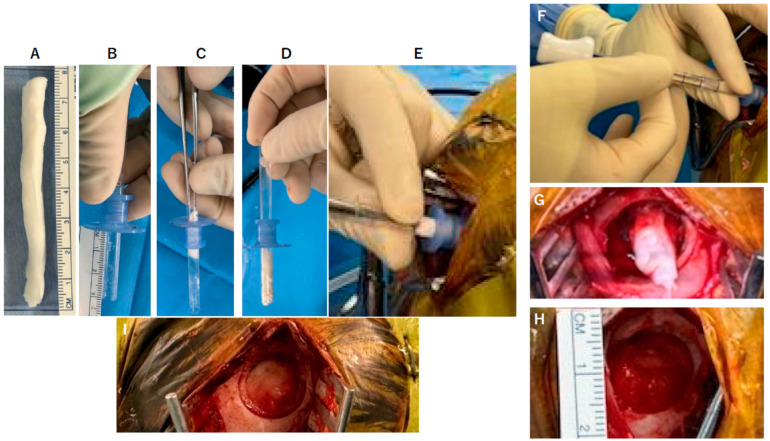
Intraoperative photographs demonstrate the technique of inserting a rolled DuraGen graft through an endoscopic NeuroPort sheath to fill a parenchymal tract. (**A**) A 7 × 7 cm^2^ DuraGen sheet is cut and rolled diagonally into a cylinder. (**B**) Pre-measurement is performed to determine the appropriate length of DuraGen needed to fill the brain parenchymal tract up to the dura. (**C**) The rolled DuraGen is inserted into the NeuroPort outer sheath. (**D**) The DuraGen roll is advanced until it reaches the tip of the NeuroPort. (**E**) The NeuroPort introducer is positioned at the planned trajectory into the brain. (**F**) While ensuring that the DuraGen does not slip out, the outer sheath of the NeuroPort is withdrawn, deploying the DuraGen in the tract. (**G**) The portion of DuraGen remaining above the dura is to be used for dural repair and is spread out over the dural surface. (**H**) The DuraGen is carefully unfurled into a flat sheet to cover the dural defect and the entire burr hole site as an onlay graft. (**I**) Final view: fibrin glue is applied over DuraGen on the surface, and the wound is closed in layers.

**Figure 2:**
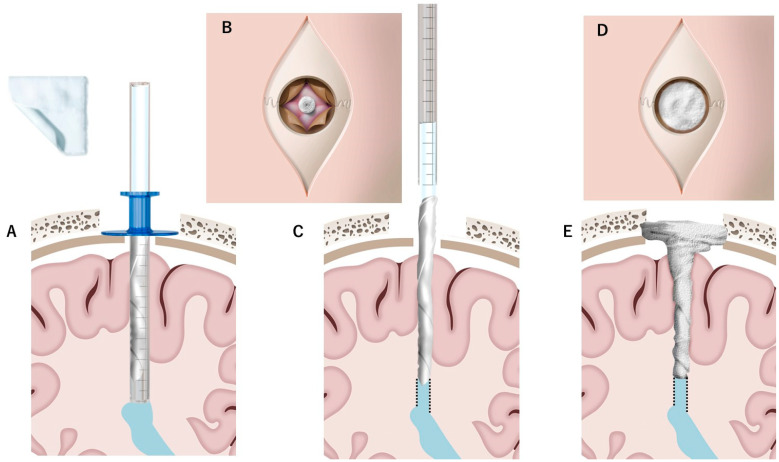
Schematic illustration of the DuraGen insertion technique shown in Figure 1. Diagrams correspond to the sequential steps (**A**–**E**) of DuraGen deployment in the parenchymal tract and over the dural defect, as described in Figure 1. The dotted line indicates the trajectory of the surgical tract created by the neuroport during the endoscopic procedure.

A 22-year-old male with hydrocephalus underwent neuroendoscopic surgery for an intraventricular lesion. Postoperative MRI showed subdural CSF accumulation along the NeuroPort sheath tract (Olympus, Tokyo, Japan), with a subcutaneous CSF collection. A 7 × 7 cm^2^ DuraGen sheet was rolled into a cylindrical form and inserted through the 12 cm-long NeuroPort outer sheath to fill the parenchymal defect without protruding into the ventricular space (Figure 1A–F and Figure 2A–C). The length of the DuraGen implant was tailored such that the proximal end of the matrix could also cover the burr hole orifice, functioning as a dural patch at the incision site (Figure 1G–I and Figure 2D,E).

In another case, Surgicel was inserted directly through the endoscopic port into the parenchymal tract using gentle compression. DuraGen and Surgicel differ not only in material composition but also in structural behavior: Surgicel^®^ rapidly absorbs fluid and loses volume, offering hemostasis but limited sealing capacity, while DuraGen maintains its scaffold properties and supports predictable absorption and tissue integration.

**Figure 3:**
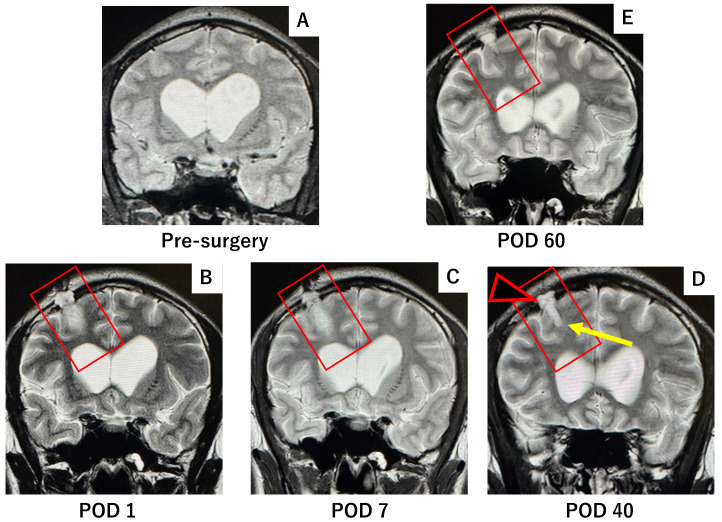
T2-weighted MRI obtained at 1, 7, 40, and 60 days postoperatively following DuraGen insertion. After neuroendoscopic surgery via the NeuroPort sheath, DuraGen was inserted into the tract. (**A**) Pre-surgery MRI before DuraGen implantation. (**B**) A faint T2-hyperintense area is observed along the surgical pathway with the DuraGen implant on postoperative day 1 (POD1). (**C**) By POD7, the hyperintensity is reduced, indicating absorption of residual cerebrospinal fluid (CSF). (**D**) At one-month post-op (POD40), the tract that contained DuraGen shows evidence of regenerating brain tissue (yellow arrow). Additionally, new dural formation is apparent at the site of the dural onlay (black arrowhead), with no CSF leakage into subdural or subcutaneous compartments. (**E**) By POD60, the parenchymal tract has largely healed, appearing comparable to preoperative imaging. Red square: Surgical field where the effect of the DuraGen® plug was assessed.

Follow-up MRI at postoperative 1, 7, 40, and 60 days demonstrated progressive resolution of the CSF leak, with the DuraGen matrix gradually absorbing and facilitating effective dural repair (Figure 3).

**Figure 4:**
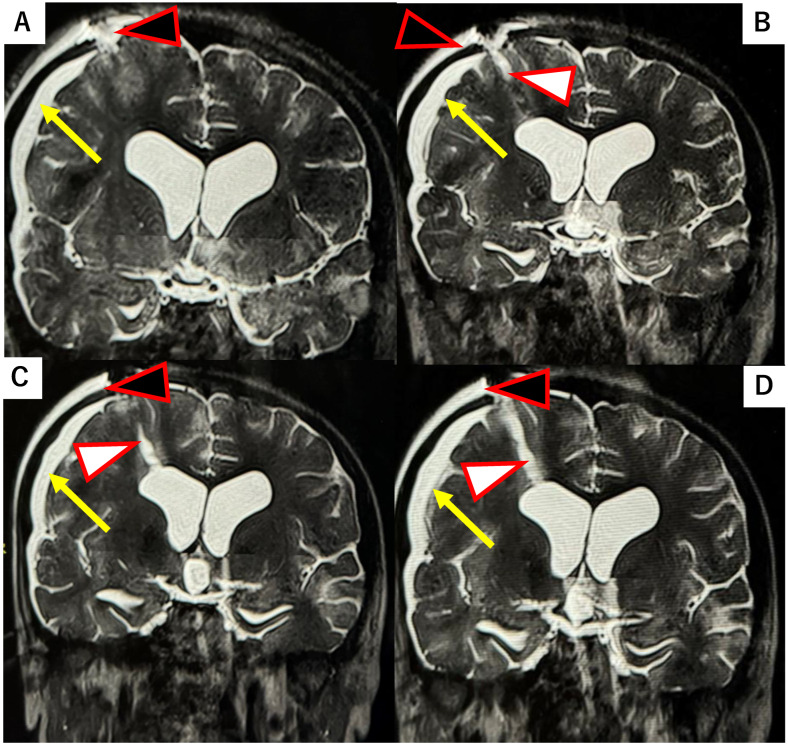
T2-weighted MRI at 30 days and two years postoperative in a case where Surgicel^®^ was used to pack the NeuroPort tract (with schematic diagram inset). (**A**–**C**) Along with the NeuroPort tract, a persistent fluid-filled parenchymal defect (white arrowhead) is visible at 30 days post-op, with reflux of CSF back into the ventricular system. This is associated with the formation of a subdural hygroma (yellow arrow). CSF accumulation is also evident in the subcutaneous tissue (effusion from the dural defect, black arrowhead). (**D**) Little to no healing of the tract is observed even at two years follow-up.

Several reports have described the use of parenchymal “plugs”, such as oxidized regenerated cellulose (Surgicel^®^; Ethicon, Inc., Bridgewater, NJ, USA) or gelatin sponge (Gelfoam®; Pfizer Inc., New York, NY, USA), to fill brain parenchymal defects [7,8]. In this case, we had the opportunity to compare the performance of Surgicel versus DuraGen in preventing CSF leaks and promoting healing, as observed on serial postoperative MRIs. Use of Surgicel was associated with persistent subdural hygroma (fluid collection) and subcutaneous fluid accumulation. In a patient where Surgicel was used to pack the NeuroPort tract, the pathway through the brain parenchyma remained as a fluid-filled defect one month postoperatively, as shown in Figure 4. In that same patient, the tract had not healed even two years postoperatively (Figure 4).

In contrast, the case using DuraGen showed no subdural or subcutaneous fluid effusion. The parenchymal defect gradually healed and was replaced by tissue of iso-intense signal to surrounding brain, with minimal granulation tissue. This regenerative process is evident in MR images at postoperative days 1, 7, 40, and 60 (Figure 3), which show the initial tract and faint high intensity along the pathway (day 1), partial absorption of CSF by day 7, and progressive parenchymal healing by days 40 and 60, along with dural restoration at the burr hole site.

### 2.2. DuraGen Implant in Porcine Brain Parenchyma

**Figure 5:**
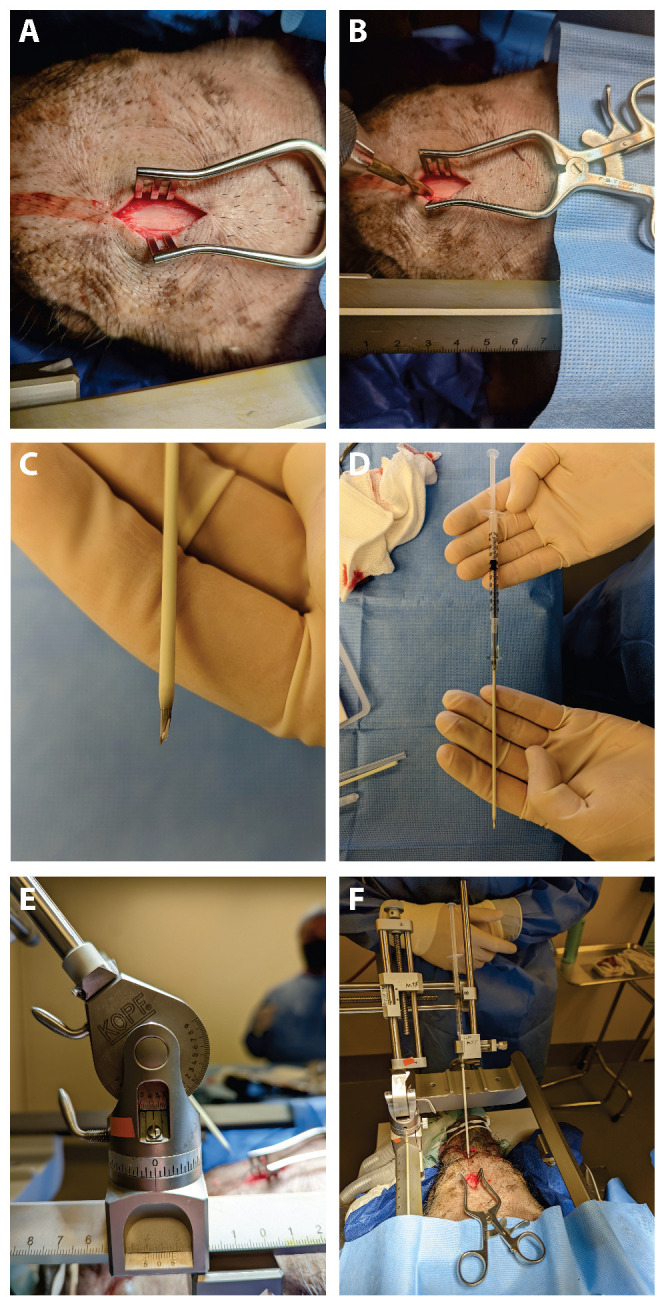
Surgical procedure for DuraGen implantation into pig brain parenchyma. (**A**) Left frontal scalp incision. (**B**) Drilling of the burr hole in the skull. (**C**) DuraGen implant visible at the tip of a loaded Angiocath needle. (**D**) Angiocath loaded with a DuraGen cylinder and clean-out wire, held in place by the syringe plunger. (**E**) The stereotactic frame holding the needle assembly in position before DuraGen deployment. (**F**) After deployment, the needle is withdrawn, leaving the DuraGen implant in the brain parenchyma (stereotactic apparatus post-deployment).

Three minipigs received DuraGen implants in one hemisphere as described in Materials and Methods, with the opposite side serving as control (Figure 5).

**Figure 6:**
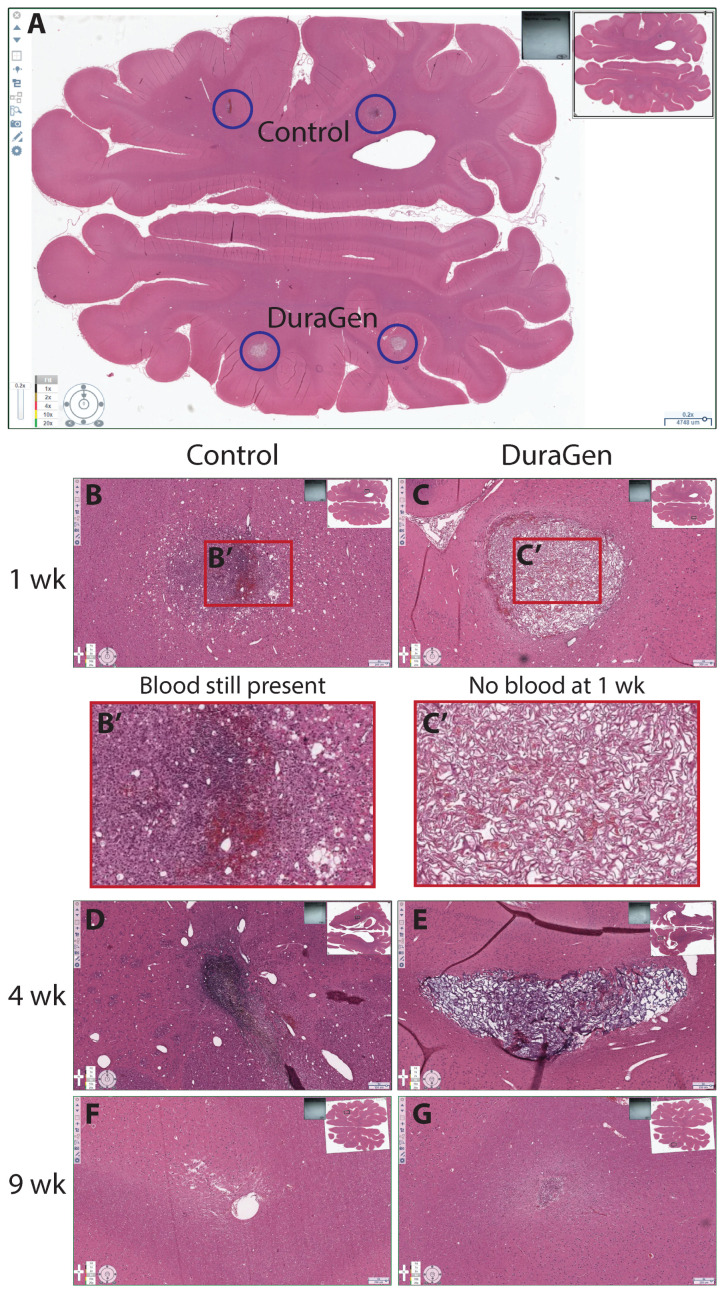
Hematoxylin and Eosin (H&E) staining of axial sections of pig brain at various time points, comparing control versus DuraGen-implanted tracts. (**A**) Whole-brain axial section (scale bar ≈ 5 mm) with circles indicating the tract locations. (**B**–**G**) Magnified views of the control tracts ((**B**,**D**,**F**); right hemisphere, no implant) and DuraGen tracts ((**C**,**E**,**G**); left hemisphere) at 1 week, 4 weeks, and 9 weeks post-surgery. Inset images of full axial brain sections indicate the location of each magnified tract image. Control tracts show more blood and cellular debris at 1 week (**B′**), while DuraGen tracts show minimal blood (**C′**) and are largely resolved by 9 weeks. DuraGen resorption is evident at 4 weeks (**E**) and almost complete at 9 weeks (**G**), while pathology in the Control tract at 4 weeks (**C**) appears to resolve into a CSF pocket at 9 weeks (**F**). Scale bars = 250 µm.

**Figure 7:**
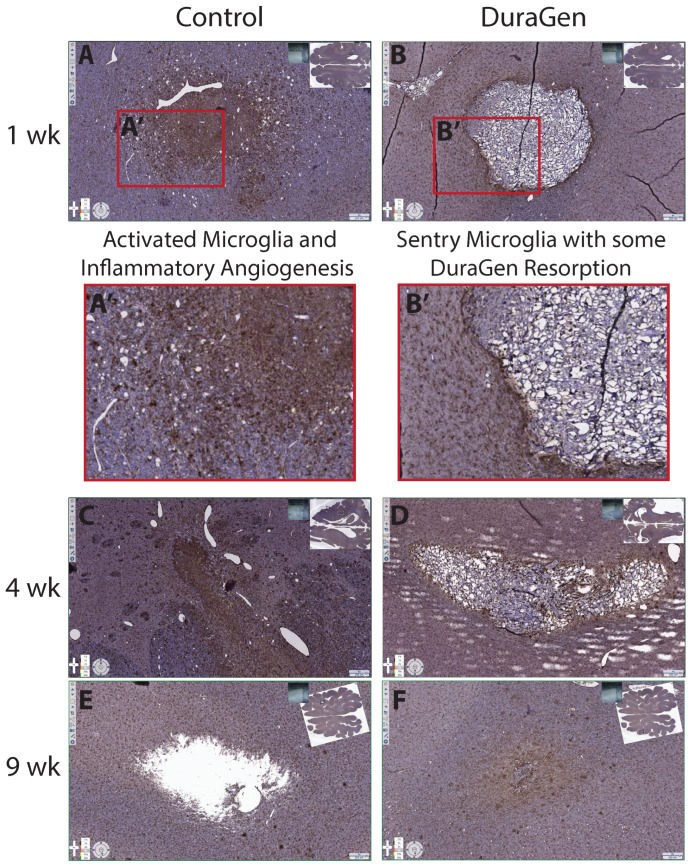
Immunohistochemical staining for ionized calcium-binding adapter molecule 1 (IBA1) (marker for microglia) showing control vs. DuraGen tracts at 1, 4, and 9 weeks. Inset images of full axial brain sections indicate the location of each magnified tract image. The control tracts (**A**,**C**,**E**) exhibit a high density of IBA1-positive activated microglial cells infiltrating the tract area (Brown DAB stain; (**A′**) and inflammatory angiogenesis surrounding the tract (circular white holes are new blood vessels), whereas the DuraGen tracts (**B**,**D**,**F**) show non-inflammatory sentry microglia with minimal activation at the periphery of the DuraGen implant (phagocytic resorption) and no inflammatory angiogenesis (**B′**). Microglial resorption of DuraGen at 4 and 9 weeks is evident in (**D**,**F**), while persistent inflammation at 4 weeks in the Control tract (**C**) is followed by a tissue gap at 9 weeks (**E**) likely filled with CSF. Scale bars = 250 µm.

This pilot study, with one animal per time point, was intended to test the implantation technique and evaluate the tissue and immune response to intraparenchymal DuraGen. We initially hypothesized that the presence of DuraGen would not impede healing or increase local inflammation, therefore we sought to compare implant tracts containing DuraGen to tracts without an implant following a natural healing trajectory. We anticipated similar host responses in control versus DuraGen tracts if the collagen were inert, indicating no safety concerns for intraparenchymal use. Instead, we found notable differences: the control tracts (no implant) showed more blood and debris on Hematoxylin and Eosin (H&E) staining (Figure 6) and a higher degree of microglial infiltration in the tract on ionized calcium-binding adapter molecule 1 (IBA1) staining compared to the DuraGen-implanted tracts (Figure 7). 

**Figure 8:**
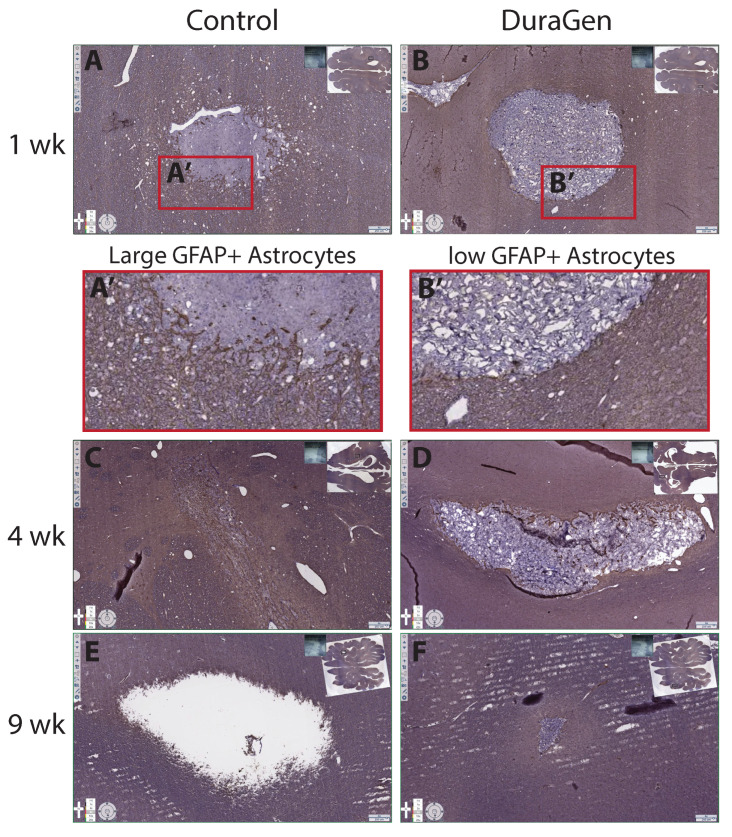
Immunohistochemical staining for glial fibrillary acidic protein (GFAP) (marker for astrocytes/glial scarring) showing control vs. DuraGen tracts at 1, 4, and 9 weeks. Insets display full-section images to indicate tract locations. The control tracts (**A**,**C**,**E**) demonstrate more pronounced astrocytosis (dense GFAP staining; (**A′**)), while the DuraGen tracts (**B**,**D**,**F**) show minimal reactive astrocytosis (**B′**). No astrocytosis is evident around the DuraGen at 4 or 9 weeks (**D**,**F**), while a glial scar appears to be present at 4 weeks in the Control tract (**C**) followed by a tissue gap at 9 weeks (**E**) likely filled with CSF. Scale bars = 250 µm.

More pronounced astrocytosis was also evident in control tracts with glial fibrillary acidic protein (GFAP) staining (Figure 8, control panels). 

At 1-week post-surgery, there was evidence of inflammatory-driven angiogenesis around the control tract, and by 9 weeks a void space (presumed CSF pocket) had formed in one control tract. In contrast, the DuraGen implant tracts had little to no residual blood or inflammation on H&E, and by 9 weeks the DuraGen material was almost entirely resorbed with no signs of damage in the surrounding parenchyma. These findings suggest that the DuraGen implant was biocompatible and may have mitigated the typical injury response in the brain tissue, in addition to simply filling the space.

## 3. Discussion

Since Nishihara et al. [1] introduced the transparent endoscopic sheath in 2000, many neurosurgeons have been able to perform neuroendoscopic procedures with greater effectiveness and safety. However, neuroendoscopic surgery is still prone to complications such as CSF leakage through the dural and parenchymal defects created by the endoscopic tract during the procedure [10,11], leading to risks like secondary meningitis [12,13] and epilepsy [10,14,15]. The treatment of an intraventricular lesion entails CSF leaks, and the tract can be packed by Surgicel or Gel foam [7,8]. Oi et al. [8] reported that subdural fluid collection occurred with Surgicel packing and a subdural-peritoneal shunt was needed in one patient. Additionally, these hemostats are often reported with inflammatory morbidities induced by granuloma formation or foreign body reaction [16,17]. Peretta et al. [11] analyzed complications in neuroendoscopic surgery, categorizing them into vascular, neural, and technical failures, and emphasized the risks of arterial bleeding, neural structure damage, and CSF leakage, highlighting that proper patient selection, meticulous technique, and intraoperative monitoring are key to minimizing adverse outcomes. In our study, we demonstrated that a properly applied DuraGen collagen matrix can effectively prevent CSF leakage through the parenchymal and dural tract. Its ability to prevent CSF leakage is corroborated by many reports, even in high-pressure scenarios such as transsphenoidal or infratentorial surgeries [18,19,20,21,22].

DuraGen, a highly purified type I collagen matrix derived from bovine deep flexor tendon [23], absorbs blood and cerebrospinal fluid immediately upon placement, forming a fibrin clot that serves as both a biological and mechanical seal against CSF leakage. The collagen matrix has an optimal pore size to serve as a scaffold for fibroblast infiltration by about 3–4 days post-surgery [24,25,26], producing new collagen with ingrowth of capillaries by 14 days [27,28]. Over 6–8 weeks, the implant is gradually resorbed and replaced by new dural tissue, as demonstrated in both clinical and experimental studies [22,23,29,30,31,32].

Our method shows promise in reducing postoperative complications across a range of neurosurgical cases involving creation of a parenchymal tract with an endoscope. The rolled DuraGen insertion technique minimized postoperative CSF leakage, potentially reducing the risk of meningitis and secondary epilepsy due to persistent meningeal irritation [10]. This approach also appears to promote dural regeneration at the defect site, which is crucial for long-term optimal outcomes. Many reports have noted an absence of inflammation, foreign-body reaction, or encapsulation in the clinical use of DuraGen [24,25,27,33,34,35,36], which is especially important for preventing chronic adhesive arachnoiditis, other inflammatory complications, and scar-related adhesions in the subarachnoid space [21,22,25,27,29,30,37,38,39,40].

Compared to previous patients in whom Surgicel were used to fill the brain tract, the current patient treated with the DuraGen plug showed no evidence of subcutaneous or extradural fluid collection. In the Surgicel case, minor subcutaneous fluid accumulation was observed postoperatively, requiring conservative monitoring.

Collagen has long been used as an inert, protective biomaterial in surgical applications [27,34], and its efficacy in reducing postoperative scar formation and fostering regenerative healing of tissue has been indicated in prior studies [32,41]. Several experimental studies have highlighted DuraGen’s versatility in supporting neural regeneration. For instance, Rabinowitz et al. [42] found that DuraGen has no adverse effect on the survival or process outgrowth of rat cortical neurons in vitro, suggesting it is a safe and effective dural substitute that does not inhibit neural growth. Finch et al. [43] demonstrated the ability of reformulated DuraGen Plus™ to support the growth and differentiation of neuronal stem cells, indicating its potential use as a scaffold for protected cell therapy in the central nervous system. Petrov et al. [41] have described how extracellular matrix materials, including collagen, interact with neural cells and tissues as scaffolds for neural repair, advancing the field of restorative and regenerative neurosurgery. Recently, the positive angiogenic effect facilitated by DuraGen in a rat model of chronic cerebral hypoperfusion was reported by Kameno et al. [44] in which indirect bypasses were created to restore perfusion using either temporalis muscle, or DuraGen. Both interventions demonstrated an equivalent increase in the ratio of cortical vascular endothelial cells. Maeda et al. [45], in their clinical study, have also reported the angiogenic effect of the DuraGen matrix, with the formation of capillaries within the newly formed dura following onlay duraplasty. The effective support of angiogenesis within DuraGen may contribute to accelerated parenchymal healing [22].

We conducted a small pilot study in pigs for preclinical feasibility testing due to their relatively large gyrencephalic brains, allowing for application of clinical techniques and form factors, and providing a translationally relevant brain environment for mechanistic study. We initially hypothesized that the presence of DuraGen would not impede healing or increase local inflammation, therefore we sought to compare implant tracts containing DuraGen to tracts without an implant following a natural healing trajectory. In our porcine model, we observed that the natural course of healing without a DuraGen implant included blood still present at 1 week, filling the parenchymal void space of the angiocatheter tract with inflammatory microglia at 4 weeks with clear signs of inflammatory angiogenesis, astrocytosis at the lesion perimeter, and eventually a tissue gap (likely filled with CSF) at 9 weeks. We were surprised to find that the DuraGen implant tracts had minimal inflammation or hemorrhage, and the collagen implant was almost completely resorbed by 9 weeks with no damage to surrounding tissue. This finding suggests that DuraGen may exert protective, anti-inflammatory, and pro-regenerative effects on the brain parenchyma, opening up new possibilities for therapeutic applications. These findings will need to be confirmed in follow-up studies in this model.

From our clinical case, we also observed that DuraGen appears to promote healing not only of dural defects but also of the parenchymal tract itself. Over time, the tract filled with DuraGen demonstrated tissue regeneration on MRI, whereas a similar tract filled with a hemostatic agent did not heal. Additional clinical cases and perhaps controlled trials will be necessary to corroborate DuraGen’s role in supporting neural tissue regeneration and to fully establish the safety and efficacy of this technique in diverse neurosurgical applications.

## 4. Materials and Methods

The study was conducted in accordance with the Declaration of Helsinki and approved by the Institutional Review Board of Tokyo Women’s Medical University (protocol code 3540-R6, 24 June 2022). And Informed consent was obtained from all subjects involved in the study.

### 4.1. DuraGen Insertion Technique

The DuraGen insertion technique is detailed as follows, with images illustrating each step in Figure 1 and Figure 2:

1. Determining the Insertion Depth: Estimate the required length of the DuraGen roll based on the measured distance from the ventricular wall to the dural surface. This ensures that the DuraGen plug reaches the dural defect without protruding into the ventricular cavity (Figure 1A).
2. Preparation of Furled DuraGen: Cut a 7 × 7 cm^2^ DuraGen sheet and roll it diagonally into a cylindrical shape to facilitate insertion into the outer sheath of the NeuroPort endoscopic system and ultimately into the parenchymal tract (Figure 1B–D).
3. Insertion Technique: Lightly moisten the cylindrical DuraGen with saline and carefully introduce it into NeuroPort outer sheath using forceps. Avoid excessive hydration to prevent premature expansion of the collagen matrix, which could impede smooth insertion (Figure 1E and Figure 2A).
4. Deploying DuraGen at the Target Site: With the DuraGen positioned inside the outer sheath, advance the NeuroPort’s inner obturator (or stylet) to the target depth while gradually retracting the outer sheath, leaving the DuraGen securely deployed within the parenchymal defect (Figure 1F,G and Figure 2B,C).
5. Final Adjustment: Any excess DuraGen left above the dura is unfurled back into a flat sheet and spread over the dural opening to act as an onlay dural graft for additional closure (Figure 1H,I and Figure 2D,E).

These steps allow precise and effective deployment of DuraGen through the parenchymal tract and over the dural defect, maximizing its sealing of the defect and prevention of CSF leakage.

Surgicel, composed of oxidized regenerated cellulose, is primarily designed for hemostasis and not for maintaining tract volume or supporting dural repair. It tends to absorb fluid quickly and dissolve, which may leave gaps that become potential sites for CSF leakage or fluid collection. In contrast, DuraGen is a collagen-based matrix with a sponge-like texture that retains its shape when hydrated and can be tailored to seal both the parenchymal tract and dural defect. While this study does not present a controlled clinical trial, the comparison is based on opportunistic clinical experience. Despite the age difference between patients, we believe that the clinical outcomes observed—especially the absence of CSF leakage and excellent wound healing in the DuraGen case—reflect the superior structural and functional characteristics of the DuraGen tract-sealing approach.

This technique may offer a safer and more predictable method for CSF leak prevention and dural reconstruction, particularly in neuroendoscopic procedures where conventional dural closure is challenging.

### 4.2. DuraGen Implantation into Pig Brain Parenchyma

All animal procedures were approved by the University of Pennsylvania Institutional Animal Care and Use Committee and performed in accordance with the Guide for the Care and Use of Laboratory Animals. We acquired three juvenile female Yucatan minipigs and randomly assigned them to one of three endpoints (1, 4, or 9 weeks). Anesthesia was induced via intramuscular ketamine and midazolam and maintained with inhaled isoflurane. Two cranial sites (one anterior and one posterior to the coronal suture) were marked for burr hole placement and implant insertion. The left hemisphere served as the treatment side with DuraGen implants, while the right hemisphere served as a control (sheath insertion without implant). Figure 5 illustrates key steps of the surgical implantation procedure: after a left frontal scalp incision (Figure 5A), a burr hole was drilled in the skull (Figure 5B). A piece of DuraGen was loaded into a 15 mm segment of a 10 gauge by 3 inch Angiocath needle assembly (Ref 382287 Beckton Dickinson, Franklin Lakes, NJ, USA; (Figure 5C). Figure 5D shows an Angiocath loaded with the DuraGen implant and a clean-out wire, held in place by the plunger of an attached syringe. In Figure 5E,F, the needle insertion apparatus is seen attached to the stereotaxic frame before and after implant deployment, respectively.

Bilateral burr holes were created with a hand drill at the marked locations. On the left (DuraGen) side, needles pre-loaded with sterile DuraGen implants were inserted stereotaxically into the brain parenchyma to a depth of 25 mm, avoiding the lateral ventricles. The implant was deployed by holding the stylet in place with a pusher arm while withdrawing the outer needle 15 mm, leaving the 15 mm DuraGen implant in the parenchyma. The needle and stylet were then fully withdrawn, leaving the collagen implant in situ. This procedure was repeated for both anterior and posterior burr holes in the left hemisphere. On the right (control) side, the Angiocath needle was inserted to the same 25 mm depth and then removed without placing any implant (the stylet was fully inserted to simulate the act of deployment). All burr holes were irrigated, and the incisions were closed with running nylon sutures.

Following surgery, animals needed to be individually housed to protect incision sites, but socialization was still available via adjacent pens and enrichment was provided via cones and balls. Buprenorphine SR was administered post-surgery for extended analgesia and animals were observed twice daily for signs of distress. No post-surgical pain or distress was observed. We determined a priori that any animals experiencing morbidity or mortality post-surgery would be excluded. No animals (0/3) were excluded from analysis.

### 4.3. Histology

Animals were euthanized at 1 week (*n* = 1), 4 weeks (*n* = 1), and 9 weeks (*n* = 1) post-implantation. Deep anesthesia was followed by transcardiac perfusion with saline for exsanguination, immediately followed by perfusion with 4% paraformaldehyde for tissue fixation. Brains were extracted and immersed in 4% paraformaldehyde overnight for complete fixation, then rinsed in phosphate-buffered saline. Each brain was sectioned in the axial (horizontal) plane to best visualize all implant tracts and surrounding tissue. Tissue blocks containing the implant sites were processed and embedded in paraffin. Using a microtome (Triangle Biomedical Sciences, Durham, NC, USA), 8 μm-thick sections were cut from each block and mounted on slides for histological staining and analysis. We performed H&E staining to assess general tissue architecture, blood infiltration, and cell morphology, as well as immunohistochemical staining for ionized calcium-binding adapter molecule 1 (IBA1) to evaluate microglial activation and glial fibrillary acidic protein (GFAP) to evaluate astrocytic reaction. These stains were examined to compare tissue response in control tracts versus DuraGen-implanted tracts over time. The analyst was not made aware of which tracts contained DuraGen, but true blinding was not possible as the collagen matrix was visible in most sections.

## 5. Limitations

It is important to state that this was a case report even though the consecutive patients showed the same features. Also, the large animal study consisted of an *n* of 1 at each time point, and while these results are compelling, they must be recognized as preliminary. Further studies with larger sample sizes will be needed to confirm whether the observed benefits are truly attributable to the DuraGen implants and to explore the mechanisms involved.

## 6. Conclusions

The tract created in the brain parenchyma by neuroendoscopic sheath insertion can serve as a conduit for postoperative CSF leak, leading to significant morbidity. We demonstrated a novel technique utilizing DuraGen in a rolled, cylindrical form to pack the parenchymal tract and seal the dural defect.

This method was effective in preventing CSF leaks and in promoting both parenchymal and dural healing. The successful outcome in our patient aligns with the histological findings from our swine model, indicating that DuraGen is biocompatible and may actively modulate the injury response to promote regeneration. This innovative approach has the potential to enhance the versatility and safety of neuroendoscopic surgery by reducing CSF leak-related complications and supporting tissue repair. Future mechanistic study in pigs and prospective studies with more patients are warranted to validate these findings and to evaluate the broader applicability of the parenchymal tract tamponade technique in neurosurgical practice.

## Data Availability

All data included in this study can be provided by contacting yaihara@twmu.ac.jp (clinical) or odj@pennmedicine.upenn.edu (preclinical).
